# Microplate bioassay to examine the effects of grapevine-isolated stilbenoids on survival of root knot nematodes

**DOI:** 10.1186/s13104-022-06106-z

**Published:** 2022-06-25

**Authors:** Christopher M. Wallis

**Affiliations:** Crop Diseases, Pests and Genetics Research Unit, USDA-ARS San Joaquin Valley Agricultural Sciences Center, 9611 S. Riverbend Ave, Parlier, CA 93648 USA

**Keywords:** Induced defense responses, Phenolics, Plant host resistance, Stilbenoids, Grapevine (*Vitis* spp.), Root knot nematodes (*Meloidogyne* spp.)

## Abstract

Root knot nematodes can be major pests in vineyards and cause significant yield losses over time. Control involves the use of different resistant grapevine rootstocks, but it remains unclear the mechanisms that such rootstocks possess to limit root knot nematode infections. Defense-associated compounds called stilbenoids, a type of phenolic compound, are present in relatively substantial amounts in grapevine root tissues. Therefore, experiments were performed to assess how different stilbenoid compounds impact nematode survival in microplate assays. Data generated were part of a larger effort to understand potential mechanisms that resistant grapevine rootstocks have to limit root knot infections. Data description: The percentage of surviving root knot nematodes was assessed 1, 3, and 5 days after J2 juveniles were placed into microplate wells amended with 0, 1.25, 2.5, 5, or 10 ppm of piceid, ε-viniferin, a resveratrol trimer putatively identified as miyabenol C, or a putative mixture of resveratrol tetramers putatively identified as vitisin B and hopeaphenol. Both ε-viniferin and the resveratrol tetramers significantly reduced root knot survival at the higher concentrations. These data provide insight about one potential mechanism that grapevine rootstocks might possess to combat nematodes.

## Objective

Root knot nematodes, *Meloidogyne incognita* and related species, can severely impact vineyard productivity where endemic and when not otherwise managed [1]. These nematodes have lifecycles whereby adult females are embedded in root tissues in “root knots”, which are specialized structures to allow full development and reproduction to occur. Due to an intimate relationship with roots, the species of grapevine (*Vitis* spp.) has a large impact on its success. Thus, many rootstocks have been selected for resistance to root knot nematodes [2–8].

However, the mechanisms of resistance remain quite unresolved. One potential source of resistance could be grapevine-produced compounds present within the roots. Such compounds could be phenolics called stilbenoids [9, 10]. Previous studies did not observe correlations between nematode counts and rootstock stilbenoids [11]. However, various factors could have impacted such results and more direct assessments were deemed warranted.

Thus, an experiment was conducted to assess stilbenoid compound effects on root knot survival. Instead of using adult nematodes, this study involved the J2 mobile stage, as it was hypothesized that stilbenoids might impact initial establishment into plant roots [4, 11]. A variety of stilbenoids were assessed, ranging from monomers to dimers to trimers to tetramers. This was done as prior work observed differences in trimers and tetramers occurred in susceptible compared to resistant grapevine rootstocks. On average, stilbenoid compounds were present around the 1 ppm level in roots, but this was highly variable [11]. Thus, a range of concentrations was utilized.

Despite results seemingly promising and significant results observed, these data only did not seem substantially enough to be published by themselves, nor were enough variety of compounds examined to justify a full report. Still, the reporting of this data could provide some useful, initial data and provide a pathway for similar studies to occur that examine the role of grapevine compounds on imparting resistance to root knot nematodes.

## Data description

These data represent the percent of J2 root knot nematodes that survived in a well over time (1, 3, or 5 days) in water amended with one of four stilbenoids at 0, 1.25, 2.5, 5, or 10 ppm. The stilbenoids included commercially-sourced (from Sigma-Aldrich, St. Louis, MO, USDA) piceid (also known as polydatin or resveratrol glycoside) or ε-viniferin (a resveratrol dimer). The compounds also included miyabenol C or vitisin B/hopeaphenol, which were isolated by high-performance liquid chromatography (HPLC) equipped with a fraction collector [a Shimadzu (Columbia, MD, USA) LC-20AD based system]. The HPLC used the same methodology of Wallis [11, 12], and compound purity was determined to be greater than 95% via HPLC-mass spectrometry (LC–MS) using the same binary solvent program. Hopeaphenol was targeted via fraction collection, but according to the follow-up LC–MS observed that the compound spontaneously formed equal amounts of two compounds with the same molecular weight of a resveratrol tetramer, one putatively identified as vitisin B and the other hopeaphenol [11].

Microplate bioassay consisted of the following. Two 96 well plates were prepared overall. This consisted of 200 µL solutions were applied to each well of one of two 96 well plates. Eight control wells had only water added. Other wells contained 1.25 ppm, 2.5 ppm, 5 ppm, or 10 ppm of stilbenoid compounds, either five wells for each concentration of the monomer piceid (resveratrol glucoside), five wells per concentration for the dimer ε-viniferin, six wells per concentration for the resveratrol trimer (putatively vitisin B, from fraction collection), or six wells per concentration for the resveratrol tetramer (putatively hopeaphenol, from fraction collection). Following solutions pipetting into each 96-well plate, everything was evaporated to dryness using a vacufuge. A suspension of an average of about 15 root knot nematodes (J2 life stage, actual range of 3–26) per 200 µL was prepared and then added to each well of the two 96-well plates to cover all treatments. 1, 3 and 5 days later, a microscope was used to count the number of live and dead root knot nematodes. For each well, percent mortality per concentration was then calculated. Univariate Analyses of Variance were used for each compound to compare survival across the four concentrations and controls using SPSS ver. 24 (IBM, Armonk, NY, USA).

Based on the statistics, ε-viniferin at 10 ppm and the stilbenoid tetramers over 2.5 ppm had root knot survival rates significantly lowered than 0 ppm controls. This suggests in this brief experiment a potential role of stilbenoid dimers and especially tetramers at limiting nematode survival. The raw data and an associated figure for day 5 counts are provided online as noted in Table 1.

**Table 1:** Overview of data files/data sets

Label	Name of data file/data set	File types (file extension)	Data repository and identifier (DOI or accession number)
Data file 1	RootKnotBioassayData	MS Excel file (.xlsx)	Ag Data Commons (https://data.nal.usda.gov/dataset/data-microplate-bioassays-examine-effects-grapevine-isolated-stilbenoids-survival-root-knot-nematodes/resource/2b2c1d45-e904-44e9-9638-51cb97d6d562) or (https://data.nal.usda.gov/system/files/RootKnotBioassayData.xlsx) [13]
Data file 2	RootKnotBioassaySummary	MS Excel file (.xlsx)	Ag Data Commons (https://data.nal.usda.gov/dataset/data-microplate-bioassays-examine-effects-grapevine-isolated-stilbenoids-survival-root-knot-nematodes/resource/ddf4ef49-40f8-412f-b9fe-89afe1788081) or (https://data.nal.usda.gov/system/files/RootKnotBioassaySummary.xlsx) [13]

## Limitations

These data consist of a small study in terms of breadth and depth. Namely, the experiment should be repeated in full to verify results, but the lack of resources had prevented this from occurring. Likewise, there are many phytocompounds worthy of testing, and the limited number of compounds tested in this bioassay suggests results be considered in that perspective. This is especially due to the fact nematodes would encounter multiple compounds at once, and not individually, but compound combinations were not examined in these data. The use of J2 juvenile root knot nematodes was necessary for practical reasons, but other life-stages should be assessed even if this would require different methodology. These bioassays also were conducted in nematodes mostly left to survive freely in water, which is very artificial and different than conditions present in natural habitats. Lastly, the use of microscopy to count nematodes could have been problematic as the field-of-view might have missed nematodes present in the wells, among other pitfalls. Despite all of this, these data could prove useful in targeting studies aiming to determine potential mechanisms of root knot resistance present in various grapevine rootstocks.

## Data Availability

The data described in this Data note can be freely and openly accessed on the National Agricultural Library’s Ag Data Commons under https://doi.org/10.15482/USDA.ADC/1524798. Please see Table 1 and Ref. [13] for details and links to the data.

## Abbreviations

HPLC: High-performance liquid chromatography
J2: The second juvenile stage of root knot nematodes which is mobile
LC–MS: Iiquid chromatography–mass spectrometry
